# The Antimicrobial Peptide Temporin G: Anti-Biofilm, Anti-Persister Activities, and Potentiator Effect of Tobramycin Efficacy Against *Staphylococcus aureus*

**DOI:** 10.3390/ijms21249410

**Published:** 2020-12-10

**Authors:** Bruno Casciaro, Maria Rosa Loffredo, Floriana Cappiello, Guendalina Fabiano, Luisa Torrini, Maria Luisa Mangoni

**Affiliations:** 1 Center For Life Nano Science@Sapienza, Istituto Italiano di Tecnologia, Viale Regina Elena 291, 00161 Rome, Italy; 2 Laboratory Affiliated to Pasteur Italia-Fondazione Cenci Bolognetti, Department of Biochemical Sciences, Sapienza University of Rome, P.le Aldo Moro 5, 00185 Rome, Italy; mariarosa.loffredo@uniroma1.it (M.R.L.); floriana.cappiello@uniroma1.it (F.C.); fabiano.1524958@studenti.uniroma1.it (G.F.); torrini.1737960@studenti.uniroma1.it (L.T.)

**Keywords:** biofilm, antimicrobial peptides, *Staphylococcus aureus*, persisters, tobramycin, drug-combination

## Abstract

Bacterial biofilms are a serious threat for human health, and the Gram-positive bacterium *Staphylococcus aureus* is one of the microorganisms that can easily switch from a planktonic to a sessile lifestyle, providing protection from a large variety of adverse environmental conditions. Dormant non-dividing cells with low metabolic activity, named persisters, are tolerant to antibiotic treatment and are the principal cause of recalcitrant and resistant infections, including skin infections. Antimicrobial peptides (AMPs) hold promise as new anti-infective agents to treat such infections. Here for the first time, we investigated the activity of the frog-skin AMP temporin G (TG) against preformed *S. aureus* biofilm including persisters, as well as its efficacy in combination with tobramycin, in inhibiting *S. aureus* growth. TG was found to provoke ~50 to 100% reduction of biofilm viability in the concentration range from 12.5 to 100 µM vs ATCC and clinical isolates and to be active against persister cells (about 70–80% killing at 50–100 µM). Notably, sub-inhibitory concentrations of TG in combination with tobramycin were able to significantly reduce *S. aureus* growth, potentiating the antibiotic power. No critical cytotoxicity was detected when TG was tested in vitro up to 100 µM against human keratinocytes, confirming its safety profile for the development of a new potential anti-infective drug, especially for treatment of bacterial skin infections.

## 1. Introduction

In nature, bacteria can alternate between a free-swimming (planktonic) life phase and a sessile phenotype called biofilm, where bacterial cells are embedded into a matrix composed of extracellular polymeric substances (EPS) consisting of exopolysaccharides, nucleic acids, proteins, lipids, and other biomolecules [1]. The EPS shields bacterial cells from the action of antibiotics, from the clearance of immune cells, and from external stressful conditions, allowing bacteria to survive in dehydrated and/or nutrient-poor media and to colonize either biological or abiotic surfaces [2,3]. Therefore, bacterial biofilms pose a serious threat to the environment and health, especially in the historical “post-antibiotic era” in which we are living. This era is characterized by a tremendous reduction in the discovery of new antibiotic drugs in parallel with an inverse emergence of resistant microorganisms [4]. Remarkably, biofilm communities contain a small subset of persister cells that enter a dormant and tolerant state to all drugs. These persister cells are often the cause of recurrent and recalcitrant biofilm-associated infections that are difficult to treat [5,6]. *Staphylococcus aureus* is one of the major human pathogens able to form biofilms and to provoke severe infections, including pulmonary, urinary, and skin infections [7]; furthermore, the emergence of clinical isolates resistant to the available antibiotics has made *S. aureus*-associated infections a serious challenge to human health [8,9]. A large US-based multicenter retrospective study conducted by Miller and coworkers showed that between 2005 and 2010 the incidence of *S. aureus*-induced skin and soft tissue infections was far higher than that of urinary tract infections or pneumonia (2.3 million cases vs 0.91 and 0.24 million cases, respectively) [10]. Moreover, from 2001 to 2009 the number of hospitalizations due to *S. aureus* skin infections increased by 123% annually, from 160,811 to 358,212 [11]. Considering the intrinsic resistance of biofilms to antibiotic therapy, the discovery of innovative approaches able to address not only planktonic bacterial cells, but also specific features of the sessile life form, is highly pressing [12]. In this context, gene-encoded antimicrobial peptides (AMPs) of the innate immunity hold promise as novel anti-biofilm agents. This is because most of them have already been found (i) to inhibit the adhesion phase of bacteria to a surface; (ii) to interfere with the matrix synthesis; (iii) to be active on metabolically-inactive cells; (iv) to possess a multimodal mechanism of bacterial killing; (v) to synergize with conventional and non-conventional antibiotics; and (vi) to display a healing capacity or immunomodulatory activity [13]. In the last decades, several AMPs with interesting biological properties have been isolated from frog-skin, one of the richest natural storehouses of AMPs. These AMPs belong to different families encompassing bombinins, brevinins, esculentins, and temporins [14]. Temporins were initially isolated from the European red frog *Rana temporaria*, and nowadays they represent one of the largest groups of amphibian AMPs (more than 100 members) [15,16] and are among the shortest AMPs (only 10-13 residues) present in nature to date. While some isoforms (i.e., temporin A, (TA [FLPLIGRVLSGIL-NH_2_]); temporin B (TB [LLPIVGNLLKSLL-NH_2_]); temporin L (TL [FVQWFSKFLGRIL-NH_2_]) of this family have already been characterized for their antimicrobial and cytotoxic properties [16,17,18,19,20,21,22,23,24], very little is known about the antimicrobial potential of temporin G (TG [FFPVIGRILNGIL-NH_2_]) [25], which was isolated from the same frog specimen, especially against the bacterial biofilm phenotype. To the best of our knowledge, this is the first manuscript describing TG activity against preformed *S. aureus* biofilm as well as against persister cells. The mechanism of the anti-biofilm activity of TG was investigated by means of fluorescence studies, while the cytotoxicity was evaluated against human keratinocytes. In addition, the ability of TG to assist the ability of the conventional antibiotic tobramycin in inhibiting microbial growth was assessed against drug-resistant *S. aureus* clinical isolates. Altogether, our results highlighted the advantageous properties of TG, either alone or in combination with tobramycin, for therapeutic treatment of skin-associated *S. aureus* infections. 

## 2. Results

### 2.1. Activity Against S. aureus Biofilm

**Figure 1:**
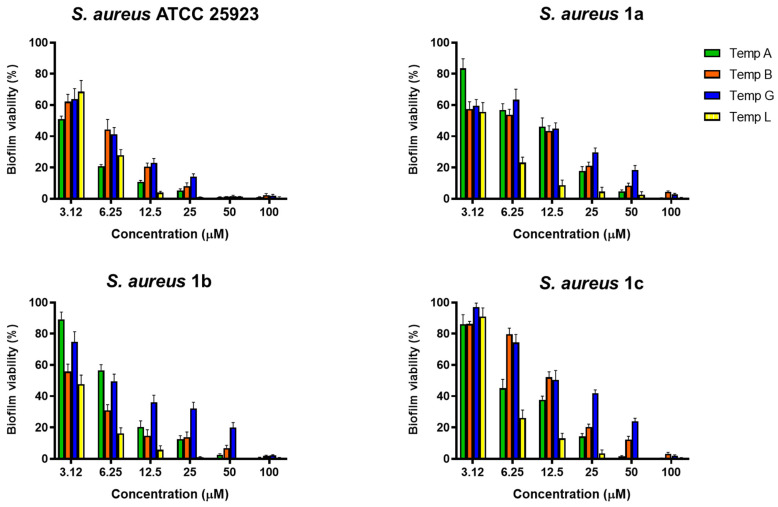
Anti-biofilm activity of TA, TB, TG, and TL against preformed biofilm of *S. aureus* ATCC 25923, *S. aureus* 1a, 1b, and 1c, after 2 h of treatment. Biofilm viability was determined as indicated in Materials and Methods and expressed as percentage compared to that of untreated samples (100%). Values are the mean of three independent experiments run in triplicate ± the standard error of the mean (S.E.M.). With the only exception of the values corresponding to the 3.12 µM against *S. aureus* 1c, all data are statistically significant (*p* < 0.05) with respect to untreated samples (100% biofilm viability).

To assess the activity of TG against preformed biofilm of *S. aureus*, the peptide was tested at different serial two-fold concentrations against the sessile form of the reference strain ATCC 25923 and drug-resistant clinical isolates namely *S. aureus* 1a, 1b, and 1c [26]. The results were compared to those of other temporin isoforms, i.e., TA, TB, and TL (Figure 1). 

All peptides showed a potent activity at 50 and 100 µM leading to ~100% killing of biofilm cells of the most sensitive *S. aureus* ATCC 25923, whereas almost total mortality of the bacterial population was caused by 100 µM of each peptide against the three clinical isolates. While the well-known TL and TA displayed the best anti-biofilm effects at all concentration ranges, TG manifested a comparable anti-staphylococcal efficacy to that of TB, despite being slightly weaker towards *S. aureus* 1b and 1c. In particular, TG was capable of killing at least 50% of *S. aureus* ATCC 25923 biofilm at 6.25 µM, whereas a concentration of 12.5 µM was necessary to provoke the same percentage of biofilm death against the three clinical isolates. 

### 2.2. Permeabilization of Biofilm Cell Membranes 

**Figure 2:**
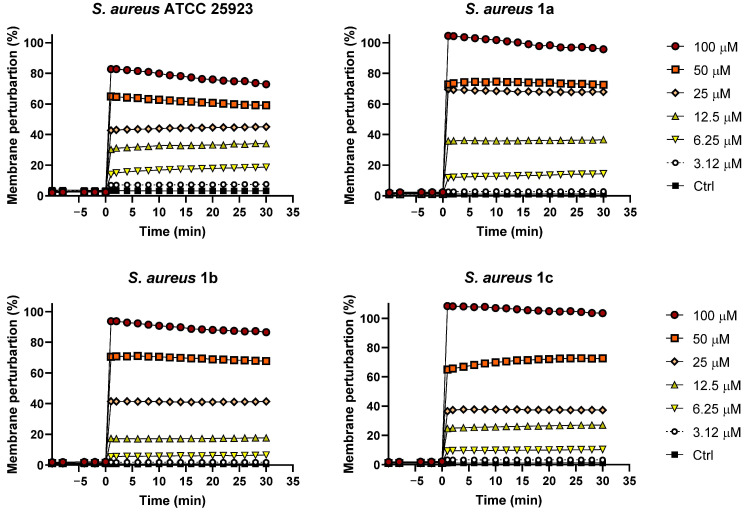
Membrane perturbation assay performed with the Sytox Green dye. The percentage of membrane damage was calculated with respect to the maximum membrane permeabilization obtained by the highest peptide concentration (100 µM) and the addition of 1 mM EDTA plus 0.5% Triton X-100. Time 0 indicates the addition of the peptide. Data points are the mean of triplicate measurements from a single experiment representative of three independent experiments. Controls (Ctrl) are cells not treated with the peptides.

Taking into account the membrane-perturbing activity of other peptide isoforms belonging to the temporin family against the planktonic form of Gram-positive bacterial strains [27], we investigated the ability of TG to perturb the membrane of *S. aureus* biofilm cells using the membrane-impermeable fluorescent Sytox Green dye. The fluorescence of this probe rapidly increases upon binding to nucleic acids, once it has entered cells with a damaged membrane. TG was analyzed at different concentrations (from 100 µM to 3.12 µM) against the biofilm form of *S. aureus* ATCC 25923 and the three clinical isolates. As reported in Figure 2, the rapid increase of fluorescence intensity after peptide addition (time 0) indicated a fast peptide-induced perturbation of biofilm cell membranes. For all the tested strains, a dose-dependent membrane-perturbing activity was obtained with ~80–100% membrane damage at 100 µM, corresponding to a concentration of TG able to completely eradicate biofilm cells (Figure 1). 

### 2.3. Activity Against Persister Biofilm Cells 

**Figure 3:**
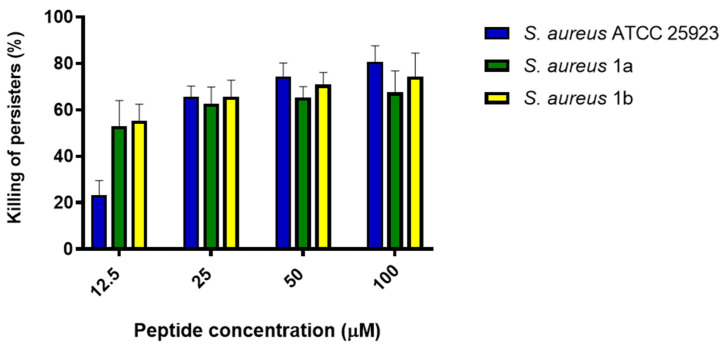
Antimicrobial activity of TG against persister biofilm cells of *S. aureus* ATCC 25923 and the clinical isolates *S. aureus* 1a and 1b after 2 h of peptide treatment. Values are the mean of three independent experiments run in triplicate ± S.E.M. All values are statistically significant (*p* < 0.05) compared to untreated samples (0% killing).

With the aim to evaluate the effect of TG against persisters, preformed *S. aureus* biofilm was treated for 24 h with a high concentration of rifampicin (i.e., 6.25 µg/mL, minimum inhibitory concentration (MIC) of 1.9 ng/mL, Table S1), as described by de Breij and colleagues [5]. Biofilm cells that survived the antibiotic action were treated with TG for 2 h, and the percentage of killing was calculated. We excluded *S. aureus* 1c, as it was resistant to rifampicin (MIC > 250 µg/mL, Table S1), the antibiotic used to obtain persister cells (see Materials and Methods). As shown in Figure 3, about 20% killing of persister biofilm cells was obtained at the lowest TG concentration (12.5 µM) against *S. aureus* ATCC 25923, while more than 50% killing was induced against the two clinical isolates. In comparison, at the higher TG concentrations (i.e., 25, 50, and 100 µM) the killing of persisters ranged from 60% to 80% against all the selected strains.

### 2.4. Effect of TG in Combination with Tobramycin in Inhibiting Bacterial Growth

**Figure 4:**
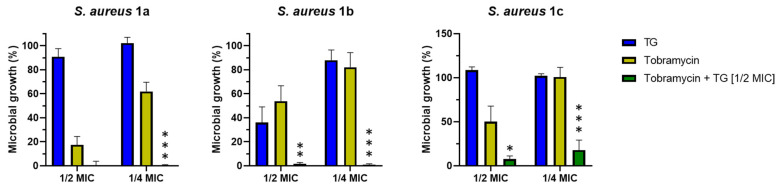
Effect of TG when used alone or in combination with tobramycin at its ½ and ¼ MIC on the growth of the three clinical isolates *S. aureus* 1a, 1b, and 1c after 20 h of peptide treatment. Values are the mean of three independent experiments run in triplicate ± S.E.M. Statistical significance between tobramycin and tobramycin plus TG (1/2 MIC) was reported as follows: * *p* < 0.05; ** *p* < 0.01; *** *p* < 0.001.

TG was also tested in combination with the commercially available and clinically-used antibiotic tobramycin in order to evaluate a possible potentiator effect in inhibiting the growth of *S. aureus* cells. As reported in Figure 4, when used at ½ MIC, TG was able to significantly enhance the efficacy of sub-inhibitory concentrations of tobramycin. More precisely, against *S. aureus* 1a, tobramycin at its ¼ MIC was able to inhibit the growth of this bacterium by only ~40%. However, when ¼ MIC of tobramycin was combined with ½ MIC of TG, the bacterial growth was completely inhibited. The same potentiator effect was observed against *S. aureus* 1b and *S. aureus* 1c. Indeed, when ½ MIC of TG was used in combination with ½ and ¼ MIC of tobramycin, the inhibitory effect of the antibiotic was significantly augmented with a total or almost complete inhibition of bacterial growth, respectively.

### 2.5. Effect on Viability of Keratinocytes 

**Figure 5:**
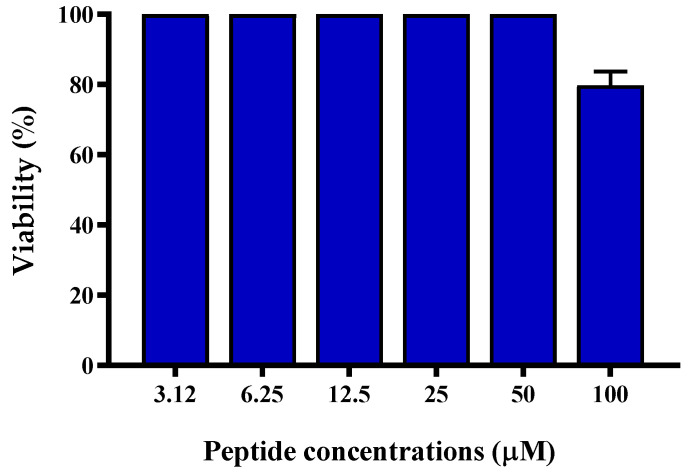
Percentage of HaCaT cell viability, as determined by the MTT assay, after exposure to increasing concentrations of TG for 24 h. Data are expressed as the mean of three independent experiments ± S.E.M. The value corresponding to 100 µM is statistically significant (*p* < 0.05) compared to the other peptide concentrations.

Considering that *S. aureus* has a principal role in the establishment of skin infections, and that keratinocytes represent the major cell type of the epidermis [28,29], the cytotoxicity of TG was evaluated by the 3-(4,5-dimethylthiazol-2-yl)-2,5-diphenyltetrazolium bromide (MTT)-based assay against the human-immortalized keratinocytes (HaCaT) after 24 h treatment with serial dilutions (from 100 µM to 3.12 µM) of peptide. Interestingly, TG was found to be totally harmless up to 50 µM, while a slight reduction in the number of metabolically-active cells (~20%) was detected at the highest concentration of 100 µM (Figure 5). Altogether, these data confirmed the safety profile of this peptide on human cells such as keratinocytes. 

## 3. Discussion

*S. aureus* is a Gram-positive bacterium causing several human skin diseases due to its ambiguous mechanisms providing protection from the host immune system [30]. Beside the production of the EPS shield, these mechanisms include also the usage of microbial surface components that identify adhesive matrix molecules, such as fibrinogen-binding proteins (ClfA and ClfB), and bind to the host cell’s surface, starting colonization [31]. In addition, biofilm-associated *S. aureus* infections become recalcitrant to conventional antibiotics, not only because of the presence of persister cells, but also because of the multiple bacterial tolerance mechanisms, such as mutation in the oxidized guanine system or in the genes involved in the prevention of oxidative damage produced by reactive oxygen species [32]. This implies a prolonged antibiotic exposure, thus predisposing bacterial cells to developing resistance [33,34,35,36]. Furthermore, a large number of antibiotics are inactivated by enzymes present in the extracellular matrix, leading to failure of the antibiotic treatment [37,38]. Therefore, the search for alternative compounds to counteract *S. aureus* infections is in highly demand [39,40]. Temporins are a family of frog-skin AMPs whose various isoforms have already shown activity against Gram-positive bacteria [23,41,42]. For instance, TA has a MIC ranging from 4 to 16 µg/mL towards human methicillin-resistant *S. aureus* (MRSA) clinical isolates [18]; TB and different synthetic analogs inhibit the growth of various *S. aureus* strains alone or in combination with TA [22,43]. The cytotoxic TL is noticeably more potent than other temporin peptides in killing *S. aureus* [44,45] and can act in synergy with TA or TB against Gram-negative bacteria by inducing changes in the biophysical properties of the peptides (e.g., prevention of their oligomeric state) when bound to the lipopolysaccharide [46]. Regarding the sessile form, TA was recently tested against *S. aureus* preformed biofilm in a study by Paduszynska et al. [36]. After 24 h of incubation, the minimum peptide concentration able to eradicate biofilm was equal to 64 mg/L. In another recent study conducted by Xie and coworkers, temporin–GHa cloned from *Hylarana guenther* was found to disrupt 90% of *S. aureus* biofilm biomass after 24 h of treatment at 100 µM [41]. A 24 h-long treatment of *S. aureus* biofilm with two different analogs of TB (at 30 µM) was investigated by Grassi and collaborators with ~1 log (90%) decrease in the number of viable biofilm cells [47]. Here for the first time we evaluated the capability of TG to eradicate *S. aureus* biofilm already after 2 h of treatment, in contrast with the long-term exposure performed in the works cited above. Moreover, we demonstrated a concomitant peptide-induced membrane perturbation as a plausible mechanism of biofilm disruption. The anti-biofilm potency correlates with the degree of membrane permeabilization. According to the model proposed for membrane-active alpha-helical peptides [48], this likely provokes the formation of local membrane breakages/pores with resulting cell death [49]. Unlike the majority of traditional antibiotics (e.g., fluoroquinolones, aminoglycosides, and β-lactams), this mechanism does not affect a particular metabolic process of the bacterial cell. As a consequence, dormant cells, i.e., persisters, cannot resist the membranolytic action of temporins [50,51,52]. Interestingly, an analogue of TB was formerly found to have a minimum bactericidal concentration (MBC) of 3.5 µM against persister planktonic cells of *S. aureus* compared to 112 µM of the currently-used lipopeptide daptomycin or the complete inactivity of colistin [53]. For these reasons, compounds like TG, which are able not only to destroy biofilms but also to kill persisters, are of great promise (Figure 3).

Another relevant feature of AMPs is their ability to synergize with traditional drugs in order to reduce the concentrations necessary to obtain an antimicrobial activity as well as to reduce potential off-target effects of the drugs. As reported by Shang and coworkers, the combination of peptides and conventional antibiotics can be a valid option to improve the effectiveness of antimicrobial agents. For instance, they demonstrated that penicillin, ampicillin, and erythromycin synergize with a series of tryptophan-containing AMPs in inhibiting the growth of multi-drug resistant *S. epidermidis* strains [54]. Similarly, Feng et al. described synergistic activity in inhibiting microbial growth between a group of short cationic AMPs and classic antibiotics (imipenem, cefepime, levofloxacin hydrochloride, and vancomycin) against Gram-negative (*Escherichia coli*, *Klebsiella pneumoniae*, and *Pseudomonas aeruginosa*) and Gram-positive (*S. epidermidis*, *Streptococcus pneumoniae*, and *S. aureus*) bacteria [55]. In another study performed by our group, a derivative of the frog-skin AMP esculentin-1a, named Esc(1-21)-1c, was found to potentiate both killing and growth inhibitory activity of the monobactam aztreonam against *P. aeruginosa* strains [56]. Here for the first time, TG was tested at its sub-inhibitory concentration (½ MIC, Table S1) in combination with ½ and ¼ MIC of tobramycin. This was sufficient to break down the growth of the three resistant *S. aureus* clinical isolates (almost 0% microbial growth, Figure 4). Such an outcome is probably due to the capability of TG to increase bacterial membrane permeability, providing facilitated access of the antibiotic to the bacterial inner compartment where it can display its toxic effect [57,58]. Indeed tobramycin is an aminoglycoside, which irreversibly binds to a site on the bacterial ribosome, inhibiting protein synthesis [59].

Finally, TG was shown to be harmless against human keratinocytes at all concentrations tested with a slight cytotoxicity (~20% reduction of metabolically-active cells) at the highest active concentration (i.e., 100 µM), confirming the potential use of this peptide against resistant *S. aureus* skin infections. Other temporin isoforms, i.e., TA and TB, have already been characterized for their plausible development as anti-infective agents against *S. aureus*-induced skin infections; they were found to kill *S. aureus* internalized into HaCaT keratinocytes and to stimulate migration of these cells by the epidermal growth factor receptor-mediated signaling pathway [60]. TA was also effective in experimentally infected surgical wounds in mice, leading to a significant bacterial growth inhibition and acceleration of wound repair [61], besides exerting a chemoattractant effect for phagocytic leukocytes in vivo [62]. 

Overall, on the basis of these studies, our current data have contributed to corroborate the potential of TG for the development of novel peptide-based formulations and/or a combination drug therapy in clinical settings, including treatment of *S. aureus* biofilm-related infections such as those found in diabetic wounds.

## 4. Materials and Methods

### 4.1. Materials, Bacterial Strains, and Cell Line

TA, TB, TG, and TL were purchased from Biomatik (Wilmington, DE, USA). Unless specifically indicated, all reagents and antibiotics used were purchased from Sigma-Aldrich (St. Luis, MO, USA). Stock solutions were prepared by dissolving the peptides in water at a concentration of 2 mM. For the microbiological assays, the reference strain *S. aureus* ATCC 25923 and the clinical isolates *S. aureus* 1a, 1b, 1c from nosocomial infections were used [26]. The resistance profile of these strains is reported in Table S2. For the cytotoxicity assay, the human-immortalized keratinocyte cell line, HaCaT (AddexBio, San Diego, CA, USA), was employed. The cells were maintained in Dulbecco’s modified Eagle’s medium supplemented with 4 mM glutamine (DMEMg), 10% heat-inactivated fetal bovine serum (FBS), and 0.1 mg/mL of penicillin and streptomycin, in a humidified incubator with 5% CO_2_ at 37 °C.

### 4.2. Evaluation of the Antibiofilm Activity of Temporin Isoforms

Preformed biofilm of *S. aureus* was obtained as previously reported with some modifications [63]. Microbial culture was grown at 37 °C to an optical density (OD) of 0.8 (λ = 590 nm) and then diluted to a cell density of 1 × 10^6^ colony forming units (CFUs)/mL. Aliquots of 100 µL were dispensed into the wells of a 96-multiwell plate, which was incubated for 20 h at 37 °C for biofilm formation. After the incubation time, the medium containing planktonic cells was aspirated from the wells and replaced by 150 µL of phosphate buffered saline (PBS) to remove any non-adherent cells. A PBS wash was performed twice. After washing, each well was filled with PBS supplemented with different two-fold serial dilutions of TA, TB, TL, and TG (from 100 to 3.12 µM), and the plate was then incubated for 2 h at 37 °C. After peptide treatment, the wells were rinsed twice with PBS, as indicated above, and 150 µL of MTT (0.5 mg/mL) was dispensed in each well in order to evaluate biofilm cell viability. In fact, this colorimetric assay involves the conversion of the water-soluble yellow dye MTT to the insoluble purple formazan crystals by mitochondrial dehydrogenases. A higher intensity of purple color corresponds to a higher percentage of metabolically-active cells and consequently to higher cell viability. The plate was incubated protected by light at 37 °C for 4 h, and the reaction was stopped by adding sodium dodecyl sulfate (SDS) (at a final concentration of 5% *v*/*v*). The absorbance of each well was recorded at 570 nm using a microplate reader (Infinite M200; Tecan, Salzburg, Austria), and the percentage of biofilm viability was calculated with respect to the untreated samples.

### 4.3. Evaluation of the Activity of TG Against Persister Biofilm Cells

In order to obtain persister biofilm cells, we followed the protocol described by de Breji and coworkers [5], with some modifications. Biofilm was formed, as described above, and after its formation it was treated with a high concentration of rifampicin (i.e., 6.25 µg/mL) for 24 h in Luria Bertani (LB) broth. After two washes with PBS, the persister biofilm was treated with serial dilutions (from 100 to 12.5 µM) of TG for 2 h, and the viable biofilm cells were evaluated by the MTT assay, as previously reported.

### 4.4. Evaluation of Membrane Permeabilization by the Sytox Green Assay

The membrane perturbing activity of TG on biofilm cells was conducted against the biofilm form of *S. aureus* ATCC 25923 and the three clinical isolates 1a, 1b, and 1c. Biofilm was formed as reported in Section 4.2 in 96-well microplates. Next, 150 μL of PBS supplemented with 1 μM Sytox Green was added to each well for 5 min in the dark. Subsequently, the peptide was added at the corresponding concentration, and changes in fluorescence intensity (λ exc = 485 nm, λ ems = 535 nm) caused by the binding of the dye to intracellular DNA were monitored for 30 min in the microplate reader (Infinite M200, Tecan, Salzburg, Austria) at 37 °C. The percentage of membrane perturbation was calculated with respect to the maximum perturbation obtained after treating bacteria with the highest peptide concentration (100 μM) and the addition of 1 mM EDTA pus 0.5% Triton X-100 (final concentration) to dissolve the EPS and make the bacterial membranes fully permeable [64]. TG was tested at concentrations ranging from 100 to 3.12 μM, and controls were cells not treated with the peptides.

### 4.5. Evaluation of the Potentiator Effect of TG by the Checkerboard Titration Assay

The checkerboard titration assay was conducted as previously reported [56] with some modifications. Combinations of TG and tobramycin in a serial two-fold dilution were added to the wells of a 96-well plate containing 1 × 10^5^ CFU/well of *S. aureus* 1a, 1b, or 1c suspended in MH. The plates were incubated for 20 h at 37 °C under mild agitation. After 20 h, absorbance (λ = 590 nm) was measured using the microplate reader. The percentage of microbial growth was calculated with respect to the untreated samples, and MIC was defined as the concentration of compound able to reduce at least 90% of the bacterial growth. MIC values are reported in Table S1. 

### 4.6. Evaluation of Peptide Cytotoxicity

To assess the cytotoxicity of TG, the amount of metabolically-active HaCaT cells was quantified by the MTT assay. Specifically, HaCaT cells were suspended in DMEMg and 2% FBS without antibiotic and plated in triplicate wells of a 96-well plate (4 × 10^4^ cells/well). After overnight incubation at 37 °C and 5% CO_2_, the medium was removed, and DMEMg containing TG at the indicated concentrations was added to each well. Cells not treated with the peptide were used as controls. After 24 h of incubation at 37 °C and 5% CO_2_, the medium was replaced by Hank’s buffer containing 0.5 mg/mL of MTT. The plate was incubated for 4 h; thereafter, formazan crystals were dissolved by adding acid-isopropanol and quantified by measuring the absorbance of each well at 570 nm using the same microplate reader. The number of metabolically-active cells was expressed as percentage compared to that of untreated control cells (100%).

### 4.7. Statistical Analysis

Unless otherwise specified, all experiments were performed three times, and the obtained values were reported as the mean ± S.E.M. When reported, statistical significance was determined by Student’s t-test using PRISM software (GraphPad, San Diego, CA, USA). *p* values of <0.05 were assumed to be statistically significant and are indicated in the legend to figures. 

## Supplementary Materials

Supplementary materials can be found at https://www.mdpi.com/1422-0067/21/24/9410/s1.

## Abbreviations

AMPs	Antimicrobial peptides
CFU	Colony forming unit
DMEM	Dulbecco’s modified Eagle’s medium
DMEMg	Dulbecco’s modified Eagle’s medium supplemented with 2 mM glutamine
EPS	Extracellular polymeric substances
FBS	Fetal bovine serum
MBC	Minimum bactericidal concentration
MIC	Mimimum inhibitory concentration
MTT	3-(4,5-dimethylthiazol-2-yl)-2,5-diphenyltetrazolium bromide
PBS	Phosphate-buffered saline

## References

[1] Batoni G., Maisetta G., Esin S. (2016). Antimicrobial peptides and their interaction with biofilms of medically relevant bacteria. Biochim. Biophys. Acta (BBA) Biomembr..

[2] Abebe G.M. (2020). The Role of Bacterial Biofilm in Antibiotic Resistance and Food Contamination. Int. J. Microbiol..

[3] Olsen I. (2015). Biofilm-specific antibiotic tolerance and resistance. Eur. J. Clin. Microbiol. Infect. Dis..

[4] Wang C.-H., Hsieh Y., Powers Z.M., Kao C.-Y. (2020). Defeating Antibiotic-Resistant Bacteria: Exploring Alternative Therapies for a Post-Antibiotic Era. Int. J. Mol. Sci..

[5] De Breij A., Riool M., Cordfunke R.A., Malanovic N., De Boer L., Koning R.I., Ravensbergen E., Franken M., Van Der Heijde T., Boekema B.K.H.L. (2018). The antimicrobial peptide SAAP-148 combats drug-resistant bacteria and biofilms. Sci. Transl. Med..

[6] Gerdes K., Semsey S. (2016). Pumping persisters. Nat. Cell Biol..

[7] Ansari S., Jha R.K., Mishra S.K., Tiwari B.R., Asaad A.M. (2019). Recent advances in Staphylococcus aureus infection: Focus on vaccine development. Infect. Drug Resist..

[8] Gajdács M. (2019). The Continuing Threat of Methicillin-Resistant Staphylococcus aureus. Antibiotics.

[9] Mermel L., Cartony J.M., Covington P., Maxey G., Morse D. (2011). Methicillin-Resistant Staphylococcus aureus Colonization at Different Body Sites: A Prospective, Quantitative Analysis. J. Clin. Microbiol..

[10] Miller N.S., Eisenberg D.F., Liu H., Chang C.-L., Wang Y., Luthra R., Wallace A.E., Fang C., Singer J., Suaya J.A. (2015). Incidence of skin and soft tissue infections in ambulatory and inpatient settings, 2005–2010. BMC Infect. Dis..

[11] Suaya J.A., Mera R.M., Cassidy A., O’Hara P., Amrine-Madsen H., Burstin S., Miller N.S. (2014). Incidence and cost of hospitalizations associated with Staphylococcus aureusskin and soft tissue infections in the United States from 2001 through 2009. BMC Infect. Dis..

[12] Pérez J., Contreras-Moreno F.J., Marcos-Torres F.J., Moraleda-Muñoz A., Munoz-Dorado J. (2020). The antibiotic crisis: How bacterial predators can help. Comput. Struct. Biotechnol. J..

[13] Browne K., Chakraborty S., Chen R., Willcox M.D., Black D.S., Parr W.C., Kumar N. (2020). A New Era of Antibiotics: The Clinical Potential of Antimicrobial Peptides. Int. J. Mol. Sci..

[14] Varga J.F.A., Bui-Marinos M.P., Katzenback B.A. (2019). Frog Skin Innate Immune Defences: Sensing and Surviving Pathogens. Front. Immunol..

[15] Simmaco M., Mignogna G., Canofeni S., Miele R., Mangoni M.L., Barra D. (1996). Temporins, Antimicrobial Peptides from the European Red Frog Rana temporaria. JBIC J. Biol. Inorg. Chem..

[16] Mangoni M.L., Di Grazia A., Cappiello F., Casciaro B., Luca V. (2015). Naturally Occurring Peptides from Rana temporaria: Antimicrobial Properties and More. Curr. Top. Med. Chem..

[17] Sikora K., Jaśkiewicz M., Neubauer D., Bauer M., Bartoszewska S., Barańska-Rybak W., Kamysz W. (2018). Counter-ion effect on antistaphylococcal activity and cytotoxicity of selected antimicrobial peptides. Amino Acids.

[18] Ciandrini E., Morroni G., Arzeni D., Kamysz W., Neubauer D., Kamysz E., Cirioni O., Brescini L., Baffone W., Campana R. (2019). Antimicrobial Activity of Different Antimicrobial Peptides (AMPs) Against Clinical Methicillin-resistant Staphylococcus aureus (MRSA). Curr. Top. Med. Chem..

[19] Jarosiewicz M., Garbacz K., Neubauer D., Kamysz W. (2020). In Vitro Efficiency of Antimicrobial Peptides against Staphylococcal Pathogens Associated with Canine Pyoderma. Anim..

[20] Paduszynska M.A., Greber K.E., Paduszynski W., Sawicki W., Kamysz W. (2020). Activity of Temporin A and Short Lipopeptides Combined with Gentamicin against Biofilm Formed by *Staphylococcus aureus* and *Pseudomonas aeruginosa*. Antibiot..

[21] Avitabile C., D’Andrea L.D., D’Aversa E., Milani R., Gambari R., Romanelli A. (2018). Effect of Acylation on the Antimicrobial Activity of Temporin B Analogues. ChemMedChem.

[22] Manzo G., Ferguson P.M., Gustilo V.B., Hind C.K., Clifford M., Bui T.T., Drake A.F., Atkinson R.A., Sutton J.M., Batoni G. (2019). Minor sequence modifications in temporin B cause drastic changes in antibacterial potency and selectivity by fundamentally altering membrane activity. Sci. Rep..

[23] Romero S.M., Cardillo A.B., Ceron M.C.M., Camperi S.A., Giudicessi S.L. (2020). Temporins: An Approach of Potential Pharmaceutic Candidates. Surg. Infect..

[24] Buommino E., Carotenuto A., Antignano I., Bellavita R., Casciaro B., Loffredo M.R., Merlino F., Novellino E., Mangoni M.L., Nocera F.P. (2019). The Outcomes of Decorated Prolines in the Discovery of Antimicrobial Peptides from Temporin-L. ChemMedChem.

[25] Mangoni M.L., Maisetta G., Di Luca M., Gaddi L.M.H., Esin S., Florio W., Brancatisano F.L., Barra D., Campa M., Batoni G. (2007). Comparative Analysis of the Bactericidal Activities of Amphibian Peptide Analogues against Multidrug-Resistant Nosocomial Bacterial Strains. Antimicrob. Agents Chemother..

[26] Casciaro B., Calcaterra A., Cappiello F., Mori M., Loffredo M.R., Ghirga F., Mangoni M.L., Botta B., Quaglio D. (2019). Nigritanine as a New Potential Antimicrobial Alkaloid for the Treatment of *Staphylococcus aureus*-Induced Infections. Toxins.

[27] Merlino F., Carotenuto A., Casciaro B., Martora F., Loffredo M.R., Di Grazia A., Yousif A.M., Brancaccio D., Palomba L., Novellino E. (2017). Glycine-replaced derivatives of [Pro 3,DLeu 9]TL, a temporin L analogue: Evaluation of antimicrobial, cytotoxic and hemolytic activities. Eur. J. Med. Chem..

[28] Bur S., Preissner K.T., Herrmann M., Bischoff M. (2013). The Staphylococcus aureus Extracellular Adherence Protein Promotes Bacterial Internalization by Keratinocytes Independent of Fibronectin-Binding Proteins. J. Investig. Dermatol..

[29] Soong G., Paulino F., Wachtel S., Parker D., Wickersham M., Zhang D., Brown A., Lauren C., Dowd M., West E. (2015). Methicillin-Resistant Staphylococcus aureus Adaptation to Human Keratinocytes. mBio.

[30] Scudiero O., Brancaccio M., Mennitti C., Laneri S., Lombardo B., De Biasi M.G., De Gregorio E., Pagliuca C., Colicchio R., Salvatore P. (2020). Human Defensins: A Novel Approach in the Fight against Skin Colonizing Staphylococcus aureus. Antibiotics.

[31] Burian M., Rautenberg M., Kohler T., Fritz M., Krismer B., Unger C., Hoffmann W.H., Peschel A., Wolz C., Goerke C. (2010). Temporal Expression of Adhesion Factors and Activity of Global Regulators during Establishment ofStaphylococcus aureusNasal Colonization. J. Infect. Dis..

[32] Miller J.H. (1996). SPONTANEOUS MUTATORS IN BACTERIA: Insights into Pathways of Mutagenesis and Repair. Annu. Rev. Microbiol..

[33] Kavanagh N., Ryan E.J., Widaa A., Sexton G., Fennell J., O’Rourke S., Cahill K.C., Kearney C.J., O’Brien F.J., Kerrigan S.W. (2018). Staphylococcal Osteomyelitis: Disease Progression, Treatment Challenges, and Future Directions. Clin. Microbiol. Rev..

[34] Suresh M.K., Biswas R., Biswas L. (2019). An update on recent developments in the prevention and treatment of Staphylococcus aureus biofilms. Int. J. Med. Microbiol..

[35] Di Pilato V., Ceccherini F., Sennati S., D’Agostino F., Arena F., D’Atanasio N., Di Giorgio F.P., Tongiani S., Pallecchi L., Rossolini G.M. (2020). In vitro time-kill kinetics of dalbavancin against Staphylococcus spp. biofilms over prolonged exposure times. Diagn. Microbiol. Infect. Dis..

[36] Ciofu O., Rojo-Molinero E., Macià M.D., Oliver A. (2017). Antibiotic treatment of biofilm infections. APMIS..

[37] Iii M.C.W., Roe F., Bugnicourt A., Franklin M.J., Stewart P.S. (2003). Contributions of Antibiotic Penetration, Oxygen Limitation, and Low Metabolic Activity to Tolerance of Pseudomonas aeruginosa Biofilms to Ciprofloxacin and Tobramycin. Antimicrob. Agents Chemother..

[38] Chiang W.-C., Nilsson M., Jensen P.Ø., Høiby N., Nielsen T.E., Givskov M., Tolker-Nielsen T. (2013). Extracellular DNA Shields against Aminoglycosides in Pseudomonas aeruginosa Biofilms. Antimicrob. Agents Chemother..

[39] Algammal A.M., Hetta H.F., Elkelish A., Alkhalifah D.H.H., Hozzein W.N., Batiha G.E.-S., El Nahhas N., Mabrok M. (2020). Methicillin-Resistant Staphylococcus aureus (MRSA): One Health Perspective Approach to the Bacterium Epidemiology, Virulence Factors, Antibiotic-Resistance, and Zoonotic Impact. Infect. Drug Resist..

[40] Grassi L., Maisetta G., Esin S., Batoni G. (2017). Combination Strategies to Enhance the Efficacy of Antimicrobial Peptides against Bacterial Biofilms. Front. Microbiol..

[41] Xie Z., Wei H., Meng J., Cheng T., Song Y., Wang M., Zhang Y. (2019). The Analogs of Temporin-GHa Exhibit a Broader Spectrum of Antimicrobial Activity and a Stronger Antibiofilm Potential against Staphylococcus aureus. Mololecules.

[42] Golda A., Kosikowska-Adamus P., Kret A., Babyak O., Wójcik K., Dobosz E., Potempa J., Lesner A., Koziel J. (2019). The Bactericidal Activity of Temporin Analogues Against Methicillin Resistant Staphylococcus aureus. Int. J. Mol. Sci..

[43] Capparelli R., Romanelli A., Iannaccone M., Nocerino N., Ripa R., Pensato S., Pedone C., Iannelli D. (2009). Synergistic Antibacterial and Anti-Inflammatory Activity of Temporin A and Modified Temporin B In Vivo. PLoS ONE.

[44] Rinaldi A.C., Mangoni M.L., Rufo A., Luzi C., Barra D., Zhao H., Kinnunen P.K., Bozzi A., Di Giulio A., Simmaco M. (2002). Temporin L: Antimicrobial, haemolytic and cytotoxic activities, and effects on membrane permeabilization in lipid vesicles. Biochem. J..

[45] Manzo G., Ferguson P.M., Hind C.K., Clifford M., Gustilo V.B., Ali H., Bansal S.S., Bui T.T., Drake A.F., Atkinson R.A. (2019). Temporin L and aurein 2.5 have identical conformations but subtly distinct membrane and antibacterial activities. Sci. Rep..

[46] Rosenfeld Y., Barra D., Simmaco M., Shai Y., Mangoni M.L. (2006). A Synergism between Temporins toward Gram-negative Bacteria Overcomes Resistance Imposed by the Lipopolysaccharide Protective Layer. J. Biol. Chem..

[47] Grassi L., Maisetta G., Maccari G., Esin S., Batoni G. (2017). Analogs of the Frog-skin Antimicrobial Peptide Temporin 1Tb Exhibit a Wider Spectrum of Activity and a Stronger Antibiofilm Potential as Compared to the Parental Peptide. Front. Chem..

[48] Dathe M., Wieprecht T. (1999). Structural features of helical antimicrobial peptides: Their potential to modulate activity on model membranes and biological cells. Biochim. Biophys. Acta (BBA) Biomembr..

[49] Sato H., Feix J.B. (2006). Peptide–membrane interactions and mechanisms of membrane destruction by amphipathic α-helical antimicrobial peptides. Biochim. Biophys. Acta (BBA) Biomembr..

[50] Lewis K. (2007). Persister cells, dormancy and infectious disease. Nat. Rev. Genet..

[51] Lewis K. (2008). Multidrug Tolerance of Biofilms and Persister Cells. Curr. Topics Microbiol. Immunol..

[52] Wood T.K. (2017). Strategies for combating persister cell and biofilm infections. Microb. Biotechnol..

[53] Grassi L., Di Luca M., Maisetta G., Rinaldi A.C., Esin S., Trampuz A., Batoni G. (2017). Generation of Persister Cells of Pseudomonas aeruginosa and Staphylococcus aureus by Chemical Treatment and Evaluation of Their Susceptibility to Membrane-Targeting Agents. Front. Microbiol..

[54] Shang D., Liu Y., Jiang F., Ji F., Wang H., Han X. (2019). Synergistic Antibacterial Activity of Designed Trp-Containing Antibacterial Peptides in Combination With Antibiotics Against Multidrug-Resistant Staphylococcus epidermidis. Front. Microbiol..

[55] Feng Q., Huang Y., Chen M., Li G., Chen Y. (2015). Functional synergy of α-helical antimicrobial peptides and traditional antibiotics against Gram-negative and Gram-positive bacteria in vitro and in vivo. Eur. J. Clin. Microbiol. Infect. Dis..

[56] Casciaro B., Loffredo M.R., Luca V., Verrusio W., Cacciafesta M., Mangoni M.L. (2019). Esculentin-1a Derived Antipseudomonal Peptides: Limited Induction of Resistance and Synergy with Aztreonam. Protein Pept. Lett..

[57] He J., Starr C.G., Wimley W.C. (2015). A lack of synergy between membrane-permeabilizing cationic antimicrobial peptides and conventional antibiotics. Biochim. Biophys. Acta (BBA) Biomembr..

[58] Kampshoff F., Willcox M.D., Dutta D. (2019). A Pilot Study of the Synergy between Two Antimicrobial Peptides and Two Common Antibiotics. Antibiotics.

[59] Goffic F., Capmau M.-L., Tangy F., Baillargé M. (1979). Mechanism of Action of Aminoglycoside Antibiotics. Binding Studies of Tobramycin and Its 6’-N-Acetyl Derivative to the Bacterial Ribosome and Its Subunits. JBIC J. Biol. Inorg. Chem..

[60] Di Grazia A., Luca V., Segev-Zarko L.-A.T., Shai Y., Mangoni M.L. (2014). Temporins A and B Stimulate Migration of HaCaT Keratinocytes and Kill Intracellular Staphylococcus aureus. Antimicrob. Agents Chemother..

[61] Simonetti O., Cirioni O., Goteri G., Ghiselli R., Kamysz W., Kamysz E., Silvestri C., Orlando F., Barucca C., Scalise A. (2008). Temporin A is effective in MRSA-infected wounds through bactericidal activity and acceleration of wound repair in a murine model. Peptides.

[62] Chen Q., Wade D., Kurosaka K., Wang Z.Y., Oppenheim J.J., Yang D. (2004). Temporin A and Related Frog Antimicrobial Peptides Use Formyl Peptide Receptor-Like 1 as a Receptor to Chemoattract Phagocytes. J. Immunol..

[63] Di Grazia A., Cappiello F., Cohen H., Casciaro B., Luca V., Pini A., Di Y.P., Shai Y., Mangoni M.L. (2015). d-Amino acids incorporation in the frog skin-derived peptide esculentin-1a(1-21)NH2 is beneficial for its multiple functions. Amino Acids.

[64] Baillie G.S. (2000). Matrix polymers of Candida biofilms and their possible role in biofilm resistance to antifungal agents. J. Antimicrob. Chemother..

