# Supplementary material for: The Antimicrobial Peptide Temporin G: Anti-Biofilm, Anti-Persister Activities, and Potentiator Effect of Tobramycin Efficacy Against Staphylococcus aureus
Source: Int J Mol Sci. 2020 Dec 10;21(24):9410. doi: 10.3390/ijms21249410 (PMC7764207; doi:10.3390/ijms21249410)
Supplement: Supplementary file 1 [file ijms-21-09410-s001.pdf]

Supplementary data

Article

The antimicrobial peptide Temporin G: anti-biofilm, anti-persister activities and potentiator effect of tobramycin efficacy against *Staphylococcus aureus*

Bruno Casciaro[1,*], Maria Rosa Loffredo[2] , Floriana Cappiello[2], Guendalina Fabiano[2], Luisa Torrini[2], Maria Luisa Mangoni[2,*]

[1] Center For Life Nano Science@Sapienza, Istituto Italiano di Tecnologia, Viale Regina Elena 291,

00161 Rome, Italy (B.C.);

[2] Laboratory affiliated to Pasteur Italia-Fondazione Cenci Bolognetti, Department of Biochemical Sciences, Sapienza University of Rome, P.le Aldo Moro 5, 00185 Rome, Italy (M.R.L, F.C., G.F., L.T., M.L.M);

* Correspondence: bruno.casciaro@iit.it, (B.C.); marialuisa.mangoni@uniroma1.it; Tel.: +39 0649910838 (M.L.M.);

Supplementary data.

Table S1. Minimum inhibitory concentrations of the tested compounds after 20 h treatment.

Strain	Rifampicin (ng/mL)	Tobramycin (μg/mL)	TG (μM)
S. aureus ATCC 25923	1.9	0.5	25
S. aureus 1a	1.9	64	12.5
S. aureus 1b	1.9	64	25
S. aureus 1c	R	32	12.5

R=resistant, >250 μg/mL

Table S2. Resistance phenotypes of the bacterial strains

Strain	Resistance phenotype[a]
S. aureus 1a	AMC-AMP-CIP-ERY-GEN-OXA
S. aureus 1b	AMC-AMP-CIP-ERY-GEN-OXA
S. aureus 1c	AMC-AMP-CIP-ERY-GEN-OXA-RIF

AMC, amoxicillin; AMP, ampicillin; CIP, ciprofloxacin; erythromycin; GEN, gentamicin; oxacillin; RIF, rifampicin.
